# Patterns of Referral to Clinical Psychology Services in the Ministry of Health Malaysia

**DOI:** 10.21315/mjms2019.26.6.11

**Published:** 2019-12-30

**Authors:** Manal Martadza, Umi Izzatti Saedon, Nooraini Darus, Tunku Saraa-Zawyah Tunku Badli, Siti Aisyah Ghazalan, Wan Mohd Azam Wan Mohd Yunus

**Affiliations:** 1 Department of Psychiatry and Mental Health, Hospital Pulau Pinang, Pulau Pinang, Malaysia; 2 Department of Psychiatry and Mental Health, Hospital Raja Perempuan Zainab II, Kota Bharu, Kelantan, Malaysia; 3 Department of Psychiatry and Mental Health, Hospital Kuala Lumpur, Kuala Lumpur, Malaysia; 4 Hospital Bahagia Ulu Kinta, Tanjung Rambutan, Perak, Malaysia; 5 Hospital Permai, Johor Bahru, Johor, Malaysia; 6 Department of Psychology, School of Human Resource Development and Psychology, Faculty of Social Sciences and Humanities, Universiti Teknologi Malaysia, Skudai, Johor, Malaysia

**Keywords:** pattern, referrals, clinical psychology, services, mental health

## Abstract

**Background:**

This descriptive study identifies the demographic characteristics and patterns of referral to clinical psychology services, which include types of diagnosis, types of referral and source of referrals in child, adolescent, adult and geriatric cases in Malaysia.

**Methods:**

We utilised 2,179 referrals between January and December 2015 from six general hospitals and three mental health institutions that provide clinical psychology services.

**Results:**

The percentage of male referrals (60.3%) is higher than that of female referrals (39.7%). Adult cases had the highest percentage of referrals (48.2%). Children (48.8%) and adolescent (28.1%) cases were mainly referred for psychological assessment. Meanwhile, adult cases (74.8%) were mainly referred for psychological intervention. Neurodevelopmental disorders was the diagnosis with the highest percentage of referrals recorded (41.4%), followed by depressive disorders (13.3%) and anxiety disorders (12.7%), and the combination of other disorders. Psychiatrists provided the highest number of referrals (82.2%), which is unsurprising as both fields are closely related.

**Conclusion:**

Clinical psychology services within the Ministry of Health (MOH) Malaysia play an important role in mental health care.

## Introduction

The demands for clinical psychology services are increasing in parallel with the growing number of patients with mental health problems. In 2006, the Australian government launched the Australian Better Access to Psychiatrists, Psychologists and General Practitioners through the Medicare Benefits Schedule (Better Access). The programme proved to be a success, with good evidence of improved access to services for common mental disorders (1). Likewise, the Blueprint II introduced by the New Zealand government also emphasised the need to have more integrated primary and secondary care services to improve mental health outcomes (2).

In Malaysia, clinical psychologists are part of a team of mental health care providers, along with psychiatrists, medical officers, counsellors and nurses. Clinical psychologists work hand in hand with psychiatrists in establishing a diagnosis and are responsible for administering and interpreting standardised psychological tests, as well as providing psychological interventions for a variety of psychiatric conditions. The National Health and Morbidity Survey estimated that 4.2 million adults are currently living with mental health problems, with an increase in the trend from 10.7% in 1996 to 29.2% of the population in 2015 (3). In relation to this, clinical psychologists play a crucial role in improving the level of mental health among Malaysians.

Nowadays, clinical psychologists in Malaysia contribute significantly beyond psychological assessments and interventions. They are actively involved in areas such as health promotion programmes, behavioural medicine, neuropsychological assessments, professional management issues and forensic sciences (4). Although very few in numbers, clinical psychologists in the Ministry of Health (MOH) Malaysia are involved in the treatment of psychologically related disorders in almost every level of sub-specialties of medicine. As such, clinical psychologists are also consistently being referred for psychologically oriented advice and recommendations for suitable treatment options by physicians, surgeons and other medical practitioners. In fact, their service in some teaching hospitals is not restricted to the psychiatric department, but they also provide services in other departments such as paediatrics, surgery, medicine, community medicine, obstetrics and gynaecology (4).

Psychiatrists are not the sole source of referrals, as shown by studies such as that by Wagner and Smith (5), who studied the referral patterns of psychological services in a paediatric clinic. Johnston (6) found that clinical psychologists in health centres received more referrals of patients with diverse problems compared with those in centrally organised services. Several studies have investigated the pattern of psychiatric referrals (7–9), but less is known about referral to clinical psychology services. This study aims to identify the demographic characteristics and patterns of referral, which include types of referral, types of diagnosis and source of referrals in children, adolescents, adults and geriatric patients.

## Subjects and Methods

A retrospective review of referral letters received by clinical psychologists from January to December 2015 was done. Data were gathered from seven clinical psychologists in five government hospitals (Hospital Pulau Pinang, Hospital Kuala Lumpur, Hospital Putrajaya, Hospital Kajang and Hospital Raja Perempuan Zainab II), five clinical psychologists from three mental institutions (Hospital Bahagia Ulu Kinta Perak, Hospital Permai Johor and Hospital Mesra Bukit Padang, Sabah) and two clinical psychologists in a rehabilitation hospital (Hospital Rehabilitasi Cheras). Data were analysed using SPSS version 22 for descriptive analysis.

## Results

A total of 2,179 referrals were received for clinical psychology services in 2015. One thousand and thirty-seven (47.6%) referrals were for assessment, 934 (42.9%) referrals were for intervention and 208 (9.5%) referrals were for both assessment and intervention. Table 1 displays the demographic characteristics and the source of referrals of the patients. Ages range from 2 to 84 years. The mean age of the study sample is 23 years.

Adult was the age group with the highest percentage of referrals for clinical psychology services (48.2%), followed by children (25.7%), adolescent (22.8%) and geriatric (3.3%). There were more male (60.3%) patients referred compared to female patients (39.7%). Patients are predominantly Malay (59.1%), followed by Chinese (22.5%), others (9.3%) and Indian (9.1%). Psychiatrists provided the highest number of referrals (82.2%).

**Figure 1:**
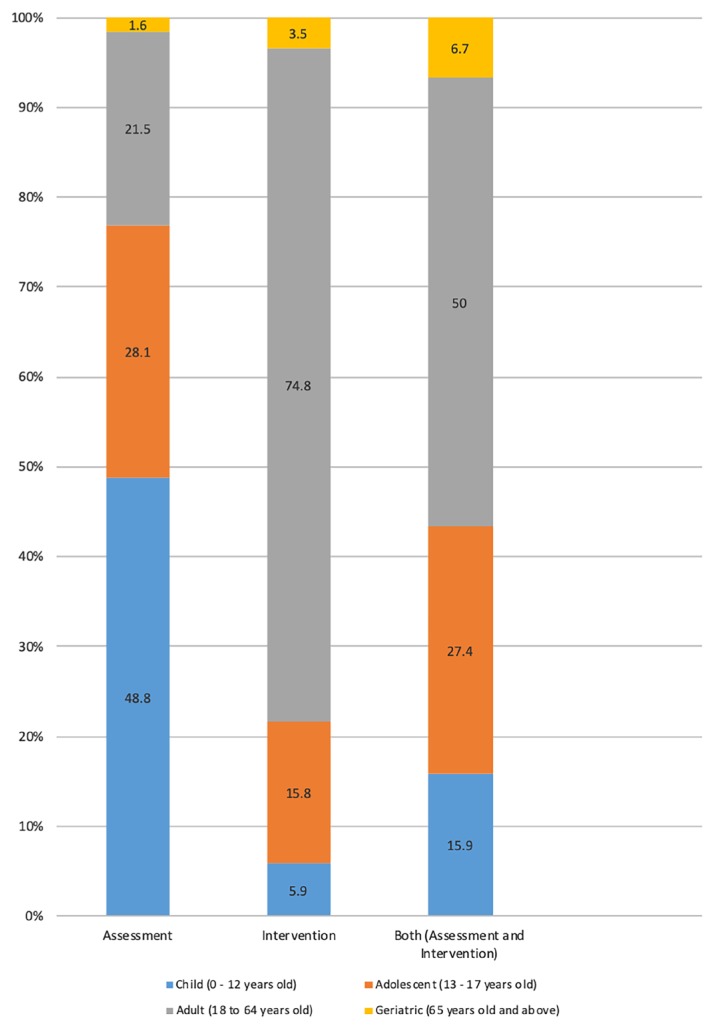
Percentage of age groups according to types of referral

Figure 1 summarises the percentage of age groups according to types of referral for clinical psychology services by age group. Psychological assessment cases were dominated by children referrals (48.8%), whereas psychological intervention cases were mainly for adult referrals (74.8%). Adult cases also recorded the highest percentage of referrals for both assessment and intervention, with half of all cases reported for this age group (50%). Notably, geriatric cases had the lowest percentages of referrals for assessment (1.6%), intervention (3.5%) or both (6.7%).

Details of the types of diagnosis are presented in Table 2. Neurodevelopmental disorders was the diagnosis with the highest percentage of referrals recorded (41.4%), followed by depressive disorders (13.4%) and anxiety disorders (12.8%). The least referred to the clinical psychology services were dissociative disorders and sexual dysfunction, with less than 0%.

**Figure 2:**
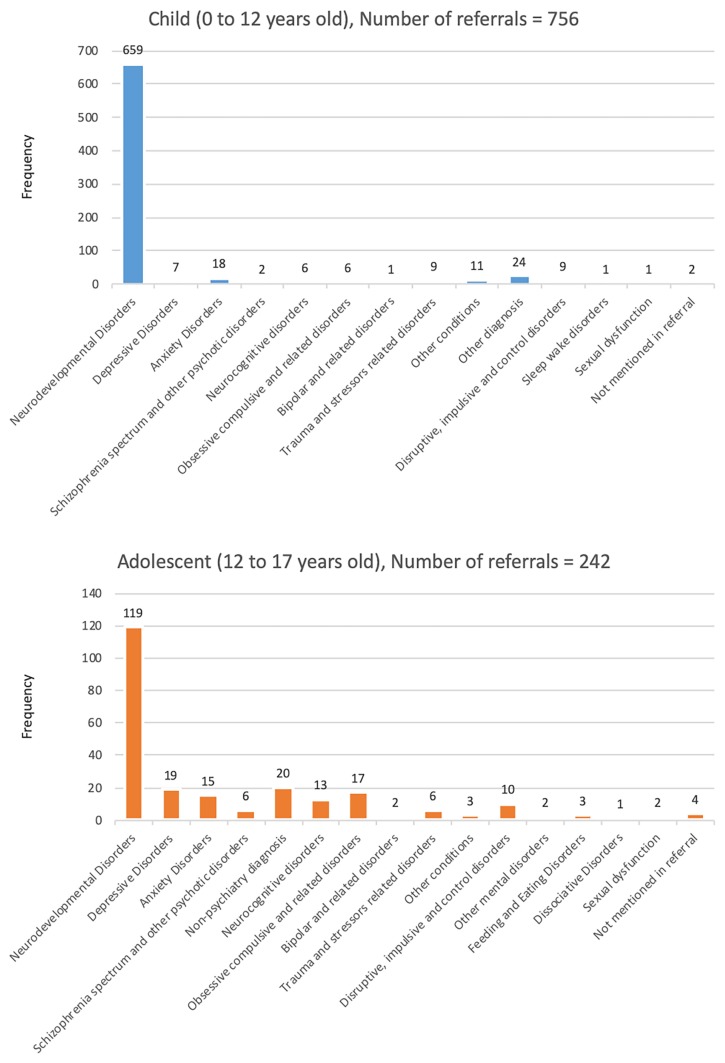
The tabulation of types of referrals (child and adolescent cases)

**Figure 3:**
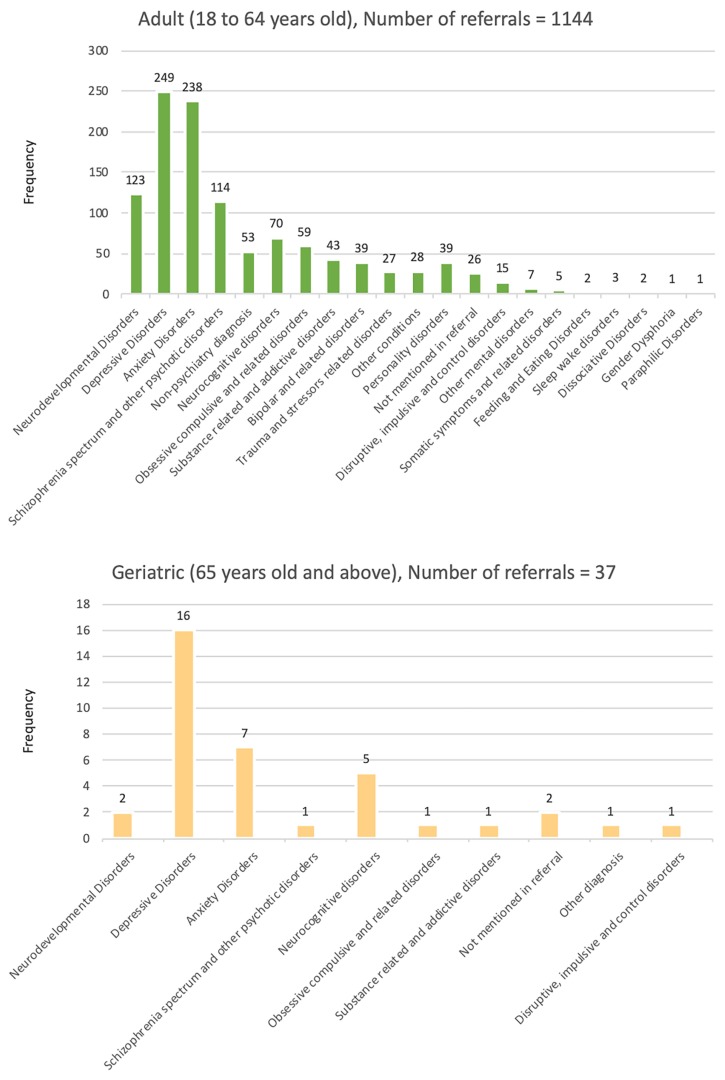
The tabulation of types of referrals (adult and geriatric cases)

Figure 2 and Figure 3 display the tabulation of the types of referral according to age groups. Similar patterns of diagnosis were seen among children (number of referrals = 659) and adolescent (number of referrals = 119), groups where majority were diagnosed with neurodevelopmental disorders, which include intellectual disabilities, autism spectrum disorders, attention deficit/hyperactivity disorder and specific learning disorders. For adults, the highest number of diagnosis is depressive disorders (number of referrals = 249), followed by anxiety disorders (number of referrals = 238). A similar pattern is seen among geriatric patients, in which they were mostly diagnosed with depressive disorders (number of referrals = 16) and anxiety disorders (number of referrals = 7).

## Discussion

Clinical psychologists in the MOH serve two major roles in patient management: evaluation of a case using standardised psychological tools and provision of psychological interventions. The range of psychological assessment to be provided includes behavioural assessment, cognitive/intelligence assessment, personality assessment and neuropsychological assessment (10). However, the types of psychological assessment provided may be subject to availability of psychological tools at different settings.

With almost half of the referrals received were for psychological assessment, clinical psychologists undeniably play a crucial role in the evaluation of patients. Psychological assessment refers to the iterative decision-making process by systematically collating data of an individual (or individuals) through multiple sources in order to adequately respond to the assessment question (11). Therefore, the understanding of psychopathology and familiarity with the Diagnostic and Statistical Manual of Mental Disorders 5 (DSM-5) are imperative as clinical psychologists and psychiatrists work hand in hand. Hence, it is not surprising that 82.2% of referrals came from psychiatrists. Psychiatrists often work collaboratively with clinical psychologists by utilising the best range of psychological assessment tools to establish and validate a diagnosis.

This has also been the practice in Australia, in which psychiatrists work with clinical and research psychologists in assessment cases, allowing the integration of information from multiple perspectives to help in understanding a particular case (12). Remaining referrers were paediatricians, anaesthesiologists and other specialists. Despite the small percentage of referrals (17.8%) from these groups, this highlights the need for clinical psychology services in non-psychiatric departments, contrary to the traditional belief that clinical psychologists only work in psychiatric settings (4). Schmaling et al. (13) pointed out that psychologists are increasingly involved with providing consultations to patients with medical illnesses, thus explaining the referrals from other specialists.

Moreover, clinical psychologists also provide appropriate psychological interventions (10). Although a consistent definition of a ‘psychological intervention’ is unclear (14), we defined a psychological intervention or treatment as non-pharmacological evidence-based practices directed towards individuals, groups, couples and families that utilise psychological theories or procedures for the treatment of mental disorders. A psychological intervention may be delivered through individual sessions or group sessions. A psychological intervention run by clinical psychologists is part of the patients’ treatment plan for psychiatric disorders, and it focuses on changing distorted behaviours, thoughts, perceptions and emotions that may be associated with specific disorders through psychological techniques, such as cognitive behaviour therapy (CBT), acceptance and commitment therapy, dialectical behaviour therapy and many other evidence-based psychological interventions. A review on the benefits of psychotherapy in 475 controlled studies showed that the condition of a typical patient after treatment was better than that of 77% of untreated controls measured at the same time, particularly for patients seeking treatment for neuroses, true phobias and emotional-somatic complaints (15).

For instance, in the Clinical Practice Guidelines, CBT is a highly recommended evidence-based treatment for major depressive disorder in Malaysia (16, 17). This could be one of the reasons why depressive disorders and anxiety disorders were the second and third highest percentages of referrals received by clinical psychologists, mainly in adult and geriatric groups. The inclusion of depressive disorders and anxiety disorders in the top three most prevalent diagnoses may be due to comorbidity. According to Strine et al. (18), comorbid depression and anxiety have been demonstrated globally. Depressed adults are 14.9 times more prone to suffer from anxiety than those without (19).

Almost half of referrals received were from children and adolescent groups (48.5%). According to the National Health and Morbidity Survey, 12.1% of the child population in Malaysia is estimated to be facing a mental health issue, especially those in the 5–9 year old group (3). This highlights the need for early intervention approaches to prevent further comorbidity and chronicity in adulthood (20). Besides general psychiatrists, child psychiatrists, speech therapists and occupational therapists, clinical psychologists in Malaysia work with a multidisciplinary team (10). In the management of children and adolescent cases, the treatment plan should be multimodal and may include psycho-education, cognitive behaviour therapy, supportive therapy, parent training and pharmacotherapy. As clinical psychologists receive training in evaluation and management in children and adolescent cases, this could be one of the factors why neurodevelopmental disorders was the diagnosis with the highest percentage of referrals (41.4%). With the rising prevalence of neurodevelopmental disorders, including attention deficit hyperactivity disorder (ADHD) (21), autism spectrum disorder (22) and intellectual disability (23), referrals to clinical psychologists are expected to increase.

The present study also revealed referrals received for male patients (60.3%) surpassing that for female patients (39.7%). Although this result is quite surprising in our settings, there is a possibility that the high percentage in male patients was influenced by the high number of referrals received for neurodevelopmental disorders. A study found that boys were more frequently referred than girls to a child psychiatry outpatient setting (24). On the other hand, the female adult population is more vulnerable to mental health issues (25) compared to their male counterparts.

## Conclusion

Clinical psychologists in the MOH play an important role in the diagnosis and treatment of children, adolescent, adult and geriatric patients, especially in psychiatry services. Children and adolescents were more often referred for psychological assessment, whereas adults were frequently referred for psychological intervention. The findings in this study are useful in delivering more efficient services suited to the needs of the population served.

## Figures and Tables

**Table 1:** Demographic characteristics and source of referrals to clinical psychology services

	Number of referrals	Percentage
Age group		
Child (0–12 years old)	561	25.7
Adolescent (13–17 years old)	496	22.8
Adult (18–64 years old)	1,050	48.2
Geriatric (65 years old and above)	72	3.3
Gender		
Male	1,315	60.3
Female	864	39.7
Ethnic		
Malay	1,288	59.1
Chinese	490	22.5
Indian	199	9.1
Others	202	9.3
Source of referrals		
Psychiatrists	1,791	82.2
Others (pain specialists, rehab specialists, pediatricians)	388	17.8

**Table 2:** Characteristics of patients referred for clinical psychology services

Type of diagnosis	Number of referrals	Percentage
Neurodevelopmental disorders	903	41.4
Depressive disorders	291	13.4
Anxiety disorders	278	12.8
Schizophrenia spectrum and other psychotic disorders	123	5.6
Non-psychiatry diagnosis	98	4.5
Neurocognitive disorders	94	4.3
Obsessive compulsive and related disorders	83	3.8
Substance related and addictive disorders	44	2.0
Bipolar and related disorders	42	1.9
Trauma and stressors related disorders	42	1.9
Other conditions that may be a focus of clinical attention	42	1.9
Personality disorders	39	1.8
Disruptive, impulsive and control disorders	35	1.6
Not mentioned in referral	35	1.6
Other mental disorders	9	0.4
Somatic symptoms and related disorders	5	0.2
Feeding and eating disorders	5	0.2
Sleep wake disorders	5	0.2
Dissociative disorders	3	0.1
Sexual dysfunction	3	0.1

Total	2179	100.0
